# Qualitative review on N‐methyl‐D‐aspartate receptor expression in rat spinal cord during the postnatal development: Implications for central sensitization and pain

**DOI:** 10.1002/dneu.22789

**Published:** 2020-11-20

**Authors:** Thomas J. de Geus, Jacob Patijn, Elbert A. J. Joosten

**Affiliations:** ^1^ Department of Anesthesiology and Pain Management Maastricht University Medical Centre Maastricht the Netherlands; ^2^ Department of Translational Neuroscience School of Mental Health and Neuroscience Maastricht University Maastricht the Netherlands

**Keywords:** central sensitization, dorsal horn, expression, neurodevelopment, NMDA‐receptor, pain

## Abstract

The N‐methyl‐D‐aspartate receptor (NMDAR) is an important mediator of central sensitization and nociception in the rat spinal dorsal horn. The NMDAR subunits and splice variants determine the properties of the receptor. Understanding the expression of NMDAR subunits in spinal cord during the neonatal development is important as it may have consequences for the process of central sensitization and nociception in later life. In this review, a systematic literature search was conducted using three databases: Medline, Embase, and PubMed. A quality assessment was performed on predetermined entities of bias. Thirteen articles were identified to be relevant. The results show that NMDAR subunits and splice variants are dynamically expressed during postnatal development in the spinal dorsal horn. During the first 2 weeks, the expression of less excitable GluN2A subunit and more sensitive GluN2B subunit increases while the expression of high excitable GluN2C subunit decreases. During the 2nd week of postnatal development GluN1 subunits with exon 21 spliced in but exon 22 spliced out are predominantly expressed, increasing phosphorylation, and transport to the membrane. The data suggest that in rats, the nociceptive system is most susceptible to central sensitization processes during the first two postnatal weeks. This may have important consequences for nociception and pain responses in later life. From this, we conclude that targeted therapy directed toward specific NMDAR subunits is a promising candidate for mechanism‐based treatment of pain in neonates.

## 1 INTRODUCTION

The dorsal horn of the spinal cord is a crucial relay station in the nociceptive pathway (Basbaum et al., 2009; Melzack & Wall, 1965). Nociceptive and non‐nociceptive afferents contact spinal neurons in the dorsal horn and this is a decision point where the nociceptive signal will be further transferred to the brain (Melzack & Wall, 1965). Nociceptive signal transduction of the communication between afferents and transmission neurons is among others regulated by N‐methyl‐D‐Aspartate receptors (NMDAR) (Basbaum et al., 2009; Kandel, 2013). The process of hypersensitivity and increased synaptic strength, is referred to as central sensitization (Basbaum et al., 2009; Latremoliere & Woolf, 2009).

Central sensitization is the enhancement of nociceptive neuron and pathway functioning. One of the mechanisms of central sensitization is strengthening of the glutamatergic synaptic connections between primary afferent and second‐order neurons. This process lowers the threshold for nociceptive signaling to pass the spinal dorsal horn and reach the brain (Latremoliere & Woolf, 2009). As the nociceptive system is still maturing during early life, external painful as well as non‐painful stimuli may affect this communication process, and may modulate the central sensitization process (Li et al., 2009; Schwaller & Fitzgerald, 2014; Van Den Hoogen et al., 2018). Increased expression and phosphorylation of postsynaptic NMDARs on the (second order) nociception‐specific transmission neurons in the spinal dorsal horn are essential in the process of central sensitization (Basbaum et al., 2009; Latremoliere & Woolf, 2009; Salter, 2005).

Within the spinal cord dorsal horn, NMDAR’s are present both presynaptic on primary afferent neurons and postsynaptic on second‐order neurons (Bardoni et al., 2004; Liu et al., 1994). Presynaptic NMDAR’s have been found to contribute to presynaptic transmitter release (Bardoni et al., 2004). Activation of postsynaptic NMDAR’s may result in action potentials and transmission of (nociceptive) signals (Basbaum et al., 2009; Kandel, 2013).

The NMDAR is composed of two GluN1 subunits combined with two other subunits, usually of the GluN2 type (GluN2A, GluN2B, GluN2C, or GluN2D subunits) (Stephenson, 2006). This very composition of the NMDAR is decisive for the nature of the function of the receptor (Ryan et al., 2008; Traynelis et al., 2010). In addition, GluN1 splice variants also determine some properties of the NMDAR including NMDA agonists/antagonists sensitivity, Mg^2+^ blockade, and potentiation by protein kinase C (Durand et al., 1993; Hollmann et al., 1993; Nakanishi et al., 1992; Sugihara et al., 1992). There are eight splice variants for the GluN1 subunit compiled from the combinations of exon splicing, including the insertion of exon 5 and deletion of exon 21 and 22 genes (Durand et al., 1993; Hollmann et al., 1993).

In the spinal dorsal horn and related to the nociceptive system, the NDMAR expresses GluN2A, GluN2B subunits (Boyce et al., 1999; Liu et al., 2004). Both subunits known to play a role in central sensitization but with different electrophysiological properties (Ji et al., 2003; Liu et al., 2004). More specific, the GluN2B incorporated NMDARs, have long‐duration excitatory postsynaptic current (EPSC) decay times, while GluN2A‐containing NMDARs have shorter decay times, thus reacting faster to a stimulus (Boyce et al., 1999; Ji et al., 2003; Liu et al., 2004). It is well known that a developmental switch from GluN2B to GluN2A expressing NMDAR’s occurs in the brain during the first two postnatal weeks (Liu et al., 2004). If a similar switch occurs in the spinal cord is unknown. Understanding the expression of NMDAR subunits in spinal cord during the neonatal development is important as it may have consequences for the process of central sensitization. A qualitative review based on a systematic search summarizing the literature on the expression of NMDAR subunits in the spinal cord during the neonatal development has never been conducted. Such a review will give more insight on the development of the nociceptive system, may show knowledge gaps that need further research, and may point toward new clinical targets for pain treatment in early life.

In this review, the normal development of the NMDAR, its subunits, and splice variants in the spinal cord is described. The data will be presented as related to different age groups based on the developmental stages of rats and humans: P0–P7, the development of the spinal cord and nociceptive system during the first postnatal week in rats is comparable to the third trimester in humans (Ian & Bryan, 2004). P7–P22, this period in rats represents the period from birth to adolescence in humans (Ohmura & Kuniyoshi, 2017). P22 > this period represents the adolescent and adulthood in humans (Ian & Bryan, 2004; Ohmura & Kuniyoshi, 2017). In rats, the (development of the) nociceptive network in the spinal dorsal horn is comparable to that of humans (Sikandar et al., 2013). In the discussion section, the expression and localization of the NMDAR subunits and splice variants in developing rat spinal cord will be linked to the function of this receptor subunit or splice variant in nociception and pain. The latter will be focused at the role of the NMDA receptor and its (clinical) implications for the process of central sensitization and pain treatment in early life.

## 2 MATERIALS AND METHODS

### 2.1 Search

A systematic search in the PubMed, Medline, and Embase databases were performed in order to select relevant articles from 1950 to June 17 2020. The search was performed for the evaluation of the NMDAR expression during the neonatal development. Search terms are described in Appendix A.

### 2.2 Selection of studies

Studies were selected based on title and abstract by two independent researchers. Full articles were read when the decision could not be formed based on the title and abstract. A consensus was made in case of disagreement or a third party was consulted for discussion.

### 2.3 Inclusion criteria

**FIGURE 1:**
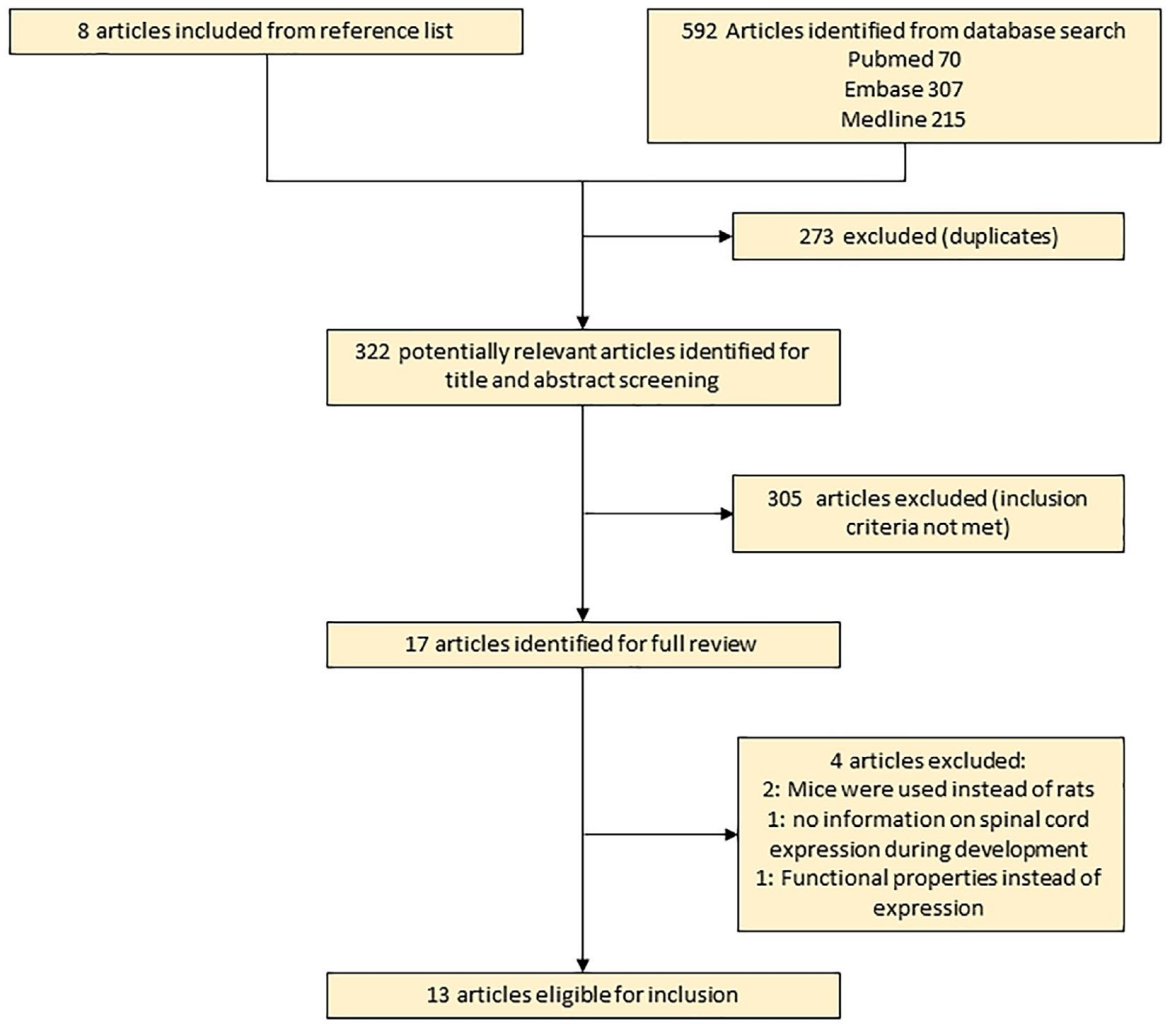
Flowchart of the study selection [Color figure can be viewed at wileyonlinelibrary.com]

Only preclinical research, published in English language, was selected. The following criteria were defined for inclusion in the expression search: the study was performed in vivo in rats, not in cell culture; Immunohistochemistry, western blotting, mRNA determination, or electron microscopy technique was used to evaluate the expression; study was performed in healthy rats, not a model for a disease of any kind (see Figure 1 for Flow‐chard).

### 2.4 Risk of bias analysis

Two authors assessed the risk of bias for each included study independently. The SYRCLE’s risk of bias tool for animal studies could not be used, as most included studies were not based on animal interventions (Hooijmans et al., 2014). Six entities for bias were assessed, including; reporting of the number of animals used, reporting of power calculation, inclusion of both genders, if applicable blinding for analysis of age groups, whether quantification was performed objective, and if the study was free of selective outcome reporting. Disagreement between authors was resolved by consensus and discussion.

### 2.5 Data extraction

Data were extracted using an extraction form. Data visualized for different time points during the postnatal development using tables.

## 3 RESULTS

### 3.1 Total NMDAR and GluN1 subunits during the neonatal development

Total NMDAR expression in the rostral (above level Th8) and caudal (below level Th8) spinal cord was quantified using western blotting (Brown et al., 2002). This quantitative analysis showed a decrease in NMDA expression in both caudal and rostral spinal cord over time, from P0 to Adult (Table 1). These results were in line with the in situ hybridization analysis of the mRNA NMDAR levels (Stegenga & Kalb, 2001) and binding assays for NMDAR agonist and antagonist (Kalb et al., 1992). NMDAR expression levels in the spinal dorsal horn do not seem to change from birth (P0) to adult (Table 1) (Brown et al., 2002; Kalb et al., 1992; Monaghan & Cotman, 1985; Stegenga & Kalb, 2001; Verhovshek et al., 2005). The decrease in total NMDAR expression is particularly notable during the first two postnatal weeks (P0–P14) and occurs within the ventral rather than the dorsal horn (Table 1) (Kalb et al., 1992). Furthermore, radio ligand binding assays confirmed this decrease in NMDAR expression in the ventral horn (Verhovshek et al., 2005). In addition, NMDAR expression levels in the spinal dorsal horn do not seem to change from birth (P0) to adult (Table 1) (Brown et al., 2002; Kalb et al., 1992; Monaghan & Cotman, 1985; Stegenga & Kalb, 2001; Verhovshek et al., 2005).

**TABLE 1:** Total NMDAR expression in spinal cord, dorsal horn, and ventral horn

	P0–P7	P7–P22	Adult
Spinal cord	++^(2,4,5)^		+^(2,4,5)^
Dorsal horn	+‐^(2)^+^(4)^	+^(2,4)^	+^(1,2,3,4,6)^
Ventral horn	++^(25)^+^(2,7)^	+‐^(2,7)^ +^(4)^	+‐^(1,4,6,7)^

Notes

(‐ undetectable; +‐ detectable; + moderate levels; and ++ high levels) (1: (Monaghan & Cotman, 1985), 2: (Kalb et al., 1992), 3: (Yung, 1998), 4: (Stegenga and Kalb, 2001), 5: (Brown et al., 2002), 6: (Nagy et al., 2004), and 7: (Verhovshek et al., 2005)).

**FIGURE 2:**
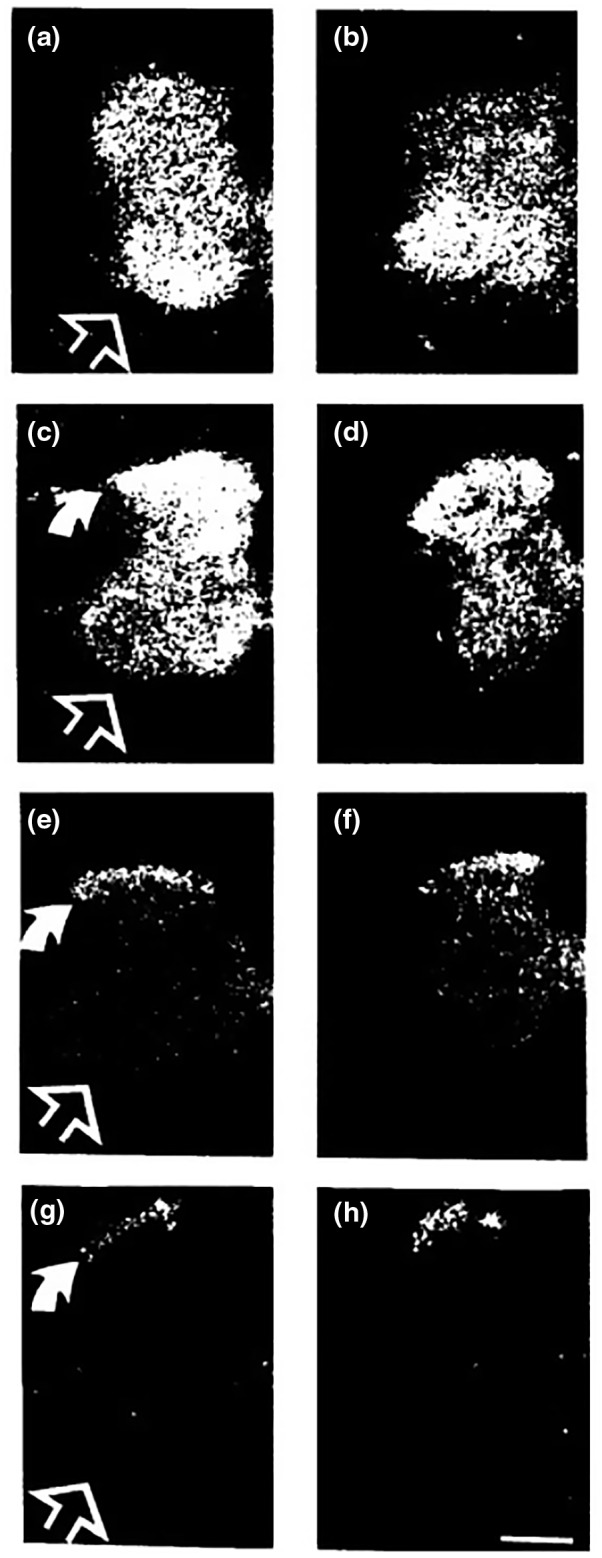
Picture and legend adapted and compressed from Kalb et al. (1992) with permission: Binding patterns of L‐[3H]glutamate for all glutamate receptors (a, c, e, and g) and NMDAR antagonist [3H]MK‐801 for binding patterns of the NMDAR‐specific (b, d, f, and h). Spinal cords from animals of various postnatal ages were labeled (P7: a,b; P14: c,d; P21:E,F; adult: g,h). P7 shows high binding of all glutamate receptors and NMDAR‐specific (a,b), including dorsal and ventral horn. At P14 the signal of both all glutamate receptor binding and NMDAR‐specific is slightly diminished throughout the spinal cord with exception of the substantia gelatinosa (Rexed lamina II) (c,d). By P21 a further decrease in both binding patterns was seen (ef). Finally, in adult rats high binding of all glutamate receptors as well as NMDAR‐specific binding was found only in the substantia gelatinosa and low levels were seen in the ventral horn. (Bar = 50 µm)

Next to the decrease in total NMDAR’s, there seems to be a change in localization within the dorsal horn (Kalb et al., 1992; Stegenga & Kalb, 2001). The abundant presence of NMDAR seen in all Rexed laminae (I–V) during the development (P0–P14) becomes more restricted to the superficial Rexed laminae (I–III) in the adult (Figure 2) (Kalb et al., 1992; Yung, 1998). Using immune‐histology higher levels of NMDAR were noted in the dorsal horn (Rexed laminae I–IV) as compared to the ventral horn in adults (Nagy et al., 2004).

Furthermore, detailed analysis of NMDA GluN1 subunit distribution in rat spinal cord during the postnatal development up till adulthood was performed using in situ hybridization (Stegenga & Kalb, 2001; Tolle et al., 1995). During the first postnatal week (P0–P7), the GluN1 expression was robust throughout the spinal cord with slightly higher staining in the dorsal horn. GluN1 signal was slightly diminished in the second postnatal week (P8–P14) (Stegenga & Kalb, 2001). The GluN1 expression was even more diminished to moderate levels in adult spinal cord, especially in the spinal dorsal horn (Stegenga & Kalb, 2001).

The expression of transcripts lacking exon 5 was moderate during the first postnatal week (P0–P7) and spread throughout the gray matter (Stegenga & Kalb, 2001). The levels of this splice varient were reduced during the second postnatal week (P7–P14). A low but detectable signal was seen in adulthood (Luque et al., 1994; Stegenga & Kalb, 2001; Tolle et al., 1995). The signal from exon 5‐containing GluN1 subunits was moderate in the first postnatal week (P0–P7). By P10 this signal was reduced and appeared higher in the dorsal horn, in adulthood the signal was low but still detectable especially in the dorsal horn (Stegenga & Kalb, 2001; Tolle et al., 1995). GluN1 subunits containing exons 21 and 22 were present at moderate levels, more abundant in the dorsal than the ventral horn during the first two postnatal weeks (P0–P14). In adulthood the levels of this splice variant were barely detectable (Luque et al., 1994; Stegenga & Kalb, 2001; Tolle et al., 1995). GluN1 subunits containing exon 21, but lacking exon 22, were highly present throughout the spinal gray matter in the first postnatal week (P0–P7), levels were slightly reduced in the second postnatal week (P7–P14) with stronger signal in the dorsal horn (Luque et al., 1994; Stegenga & Kalb, 2001; Tolle et al., 1995). In adults, the signal was low but detectable and homogeneous throughout the gray matter (Luque et al., 1994; Stegenga & Kalb, 2001; Tolle et al., 1995). Splice variants with exon 22 present, but 21 spliced out, were only detectable in the substantia gelatinosa (Rexed laminea II) in the first postnatal week (P0–P7) (Luque et al., 1994; Stegenga & Kalb, 2001; Tolle et al., 1995). GluN1 subunits without exon 21 and 22 were particularly present in the ventral rather than the dorsal horn during the first postnatal week (P0–P7), moderately present and equally distributed throughout the spinal cord gray matter in the second postnatal week (P7–P14) and low but detectable in adulthood (Luque et al., 1994; Stegenga & Kalb, 2001; Tolle et al., 1995).

Based on the five selected papers (Kalb et al., 1992; Monaghan & Cotman, 1985; Nagy et al., 2004; Stegenga & Kalb, 2001; Yung, 1998) using different techniques we can conclude that NMDAR expression levels in the spinal dorsal horn do not seem to change from Birth (P0) to Adult. At the same time, two separate papers (Kalb et al., 1992; Stegenga & Kalb, 2001) show a rearrangement of the signal for NMDAR: from total NMDAR in all Rexed laminae I–V during early development to a more restricted localization in superficial Rexed laminae I–III in the adult. With regard to GluN1 splice variants, we found one paper (Stegenga & Kalb, 2001) on the developmental expression and two papers (Luque et al., 1994; Tolle et al., 1995) reporting on the adult levels. Based on these papers we can conclude that; during the first postnatal week (P0–P7) there are low levels of all splice variants in the dorsal horn and higher levels of splice variants containing exon 22 but lacking exon 21 in the substantia gelatinosa. During the second postnatal week (P7–P14) GluN1 splice variants containing 21 but not 22 are more abundant in the dorsal horn, GluN1 splice variants contain exon 5 are more expressed in the adult dorsal horn.

### 3.2 GluN2A NMDA subunit during the neonatal development

With use of immunoblotting techniques the total amount of GluN2A protein in either rostral (above level Th8) or caudal (below level Th8) was measured (Table 2) (Brown et al., 2002). Then, in both parts of the spinal cord relatively high levels of GluN2A were detected at birth (P0), which even increased and peaked during the first two postnatal weeks (P0–P14) and then, declined toward low but still detectable levels at adulthood (Table 2) (Brown et al., 2002). These findings were partly confirmed based on in situ hybridization analysis and detection of mRNA of this GluN2A subunit (Stegenga & Kalb, 2001). High levels of GluN2A mRNA expression were reported in and around motoneurons in the ventral spinal horn at birth (P0), a signal which maintained up till P10 and then, gradually decreased (Table 2) (Stegenga & Kalb, 2001). The low levels of GluN2A transcripts were evident throughout the remaining spinal cord gray matter at birth (P0) (Table 2) (Stegenga & Kalb, 2001). At P10, the low levels of GluN2A mRNA were observed throughout the gray matter with a modest accentuation in the substantia gelatinosa (Table 2) (Stegenga & Kalb, 2001). The autoradiographic signal and thus presence of GluN2A mRNA in the adult, was very low and just above the background levels with a slightly higher signal detected in the substantia gelatinosa (Table 2) (Luque et al., 1994; Stegenga & Kalb, 2001). With use of immunocytochemical staining technique a dense staining of cell bodies was reported for GluN2A protein throughout both the dorsal Rexed laminea III–IV and ventral horn including motor neurons (Table 2) (Boyce et al., 1999; Luque et al., 1994; Nagy et al., 2004). Moreover, based on an in situ hybridization and an immunohistochemical techniques no GluN2A mRNA signal was found in both lumbar and cervical spinal cord of adult rat (Table 2) (Tolle et al., 1993; Yung, 1998).

**TABLE 2:** GLUN2A expression in spinal cord, dorsal horn, and ventral horn

	P0–P7	P7–P22	Adult
Spinal cord	++^(5)^+^(6)^	++^(6)^+^(5)^	+‐^(5,6)^‐^(1,3)^
Dorsal horn	+‐^(5)^	+^(5)^	+ ^(2,4,5,6,7)^‐^(1,3)^
Ventral horn	++^(5)^	+^(5)^	+^(4)^‐^(1,3)^

Notes

(‐ undetectable; +‐ detectable; + moderate levels; and ++ high levels) (1: (Tolle et al., 1993), 2: (Luque et al., 1994), 3: (Yung, 1998), 4: (Boyce et al., 1999), 5: (Stegenga & Kalb, 2001), 6: (Brown et al., 2002), and 7: (Nagy et al., 2004)).

Based on one developmental‐oriented paper (Stegenga & Kalb, 2001) and five papers on adult expression (Boyce et al., 1999; Brown et al., 2002; Luque et al., 1994; Nagy et al., 2004; Stegenga & Kalb, 2001) we conclude that: the GluN2A NMDA receptor is observed in demonstratable but low levels in spinal dorsal horn at birth (P0), this seems to increase to moderate levels during maturation in the first postnatal week (P0–P7) up to adulthood. Moderate levels of GluN2A NMDA receptor subunit is noted in spinal dorsal horn at P10. Moderate to high levels of GluN2A were noted in the ventral horn during the first postnatal week (P0–P7) that declines toward very moderate levels in adulthood compared to the substantia gelatinosa where a higher signal was detected at this time point.

### 3.3 GluN2B NMDA subunit during the neonatal development

Using western blotting technique, it was shown that the GluN2B is present in the spinal cord at birth (P0) (Table 3) (Brown et al., 2002). These expression levels of GluN2B seem to decline during the first two postnatal weeks (P0–P14) of neonatal development in rats (Table 3) (Brown et al., 2002). In line with the western blotting findings a decline in GluN2B expression levels were found from P0 to adulthood using in situ hybridization (Table 3) (Stegenga & Kalb, 2001). Moderate levels of GluN2B mRNA were reported in rat spinal cord dorsal horn with use of an in situ hybridization technique during the first postnatal week (P0–P7) (Table 3) (Stegenga & Kalb, 2001). Studies using an in situ hybridization and immunohistochemical analysis showed a moderate staining of GluN2B in laminae I and II of the adult dorsal horn (Table 3) (Boyce et al., 1999; Luque et al., 1994; Nagy et al., 2004; Yung, 1998). In contrast, in two different papers using an in situ hybridization technique, GluN2B mRNA levels were not detectable in neither adult spinal dorsal nor ventral horn (Table 3) (Stegenga & Kalb, 2001; Tolle et al., 1993).

**TABLE 3:** GLUN2B expression in spinal cord, dorsal horn, and ventral horn

	P0–P7	P7–P22	Adult
Spinal cord	++^(6)^ +^(5)^	+^(6)^ +‐^(5)^	+^(6)^ ‐^(1,5)^
DORSAL horn	+^(5)^	+‐^(5)^	+^(2,3,4,7)^‐^(1,5)^
Ventral horn			‐^(1)^

Notes

(‐ undetectable; +‐ detectable; + moderate levels; and ++ high levels) (1: (Tolle et al., 1993), 2: (Luque et al., 1994), 3: (Yung, 1998), 4: (Boyce et al., 1999), 5: (Stegenga & Kalb, 2001), 6: (Brown et al., 2002), and 7: (Nagy et al., 2004)).

From one developmental‐oriented paper (Stegenga & Kalb, 2001) and four papers (Boyce et al., 1999; Luque et al., 1994; Monaghan & Cotman, 1985; Verhovshek et al., 2005) on adult expression we conclude that: the GluN2B receptor is expressed in low to moderate levels in the spinal dorsal horn during the first postnatal week of development and increases to moderate levels in adulthood. Two papers (Stegenga & Kalb, 2001; Tolle et al., 1993) did not detect this subunit in the spinal dorsal horn in adults. High to moderate levels in the whole spinal cord were found during the first postnatal week by two separate articles (Brown et al., 2002; Stegenga & Kalb, 2001). These expression levels decline toward low or undetectable levels in adulthood.

### 3.4 GluN2C, GluN2D, GluN3, and GluN4 subunit during the neonatal development

With use of in situ hybridization analysis moderate levels of GluN2C mRNA were found in spinal ventral and in dorsal horn at birth (P0) (Stegenga & Kalb, 2001). Very low levels of GluN2C mRNA were reported in rat spinal cord at P10, more specific in and around spinal motor neurons in ventral horn (lamina IX), which gradually decreased till GluN2C mRNA was absent in adult spinal cord (Stegenga & Kalb, 2001; Tolle et al., 1993). Absent or very low GluN2D mRNA levels were detected in adult rat spinal cord at any time point during the postnatal development (Stegenga & Kalb, 2001; Tolle et al., 1993). Immunocytochemical analysis revealed that high levels of GluN3A subunits were present in the adult spinal dorsal horn as well as in Rexed lamina IX (motor neurons) of the adult ventral horn (Wong et al., 2002). Intense GluN3B‐immunocytochemical staining was seen in both adult spinal dorsal horn Rexed lamina I–II and the ventral horn laminae VIII–IX, moderate immunoreactivity was reported in adult dorsal horn Rexed lamina III–VI and ventral horn VII (Wee et al., 2008). Based on our search there are no research data available on presence of NMDA GluN4 subunit during development in adult spinal cord.

Based on only two articles found on GluN2C/GluN2D (Stegenga & Kalb, 2001; Tolle et al., 1993) and two on GluN3 subunits (Wee et al., 2008; Wong et al., 2002), we can conclude that: the GluN2C and GluN2D NMDA receptor subunits in the adult spinal cord are difficult to detect. GluN2C NMDA receptor subunit is present during the first two postnatal weeks. The GluN3A and GluN3B NMDA receptor subunits are abundantly present in the superficial layers of the adult spinal dorsal horn. There were no research data available on presence of GluN4 subunit.

### 3.5 Risk of bias analysis

Risk of bias was assed as described in the methods. The SYRCLE’s risk of bias tool for animal studies (Hooijmans et al., 2014) could not be used as most articles did not include treatment or interventional groups. The absence of an intervention was not exclusion criterion and treatment groups were not necessary to provide information on NMDAR subunit or spice variant expression levels during normal development. Nonetheless, this makes qualification of the articles included based on selection bias, performance bias, detection bias, attrition bias, and reporting bias not possible.

Our systematic analysis did show an absence of reporting in the number of animals in 38% (5/13) of the articles used in the study (Boyce et al., 1999; Kalb et al., 1992; Luque et al., 1994; Monaghan & Cotman, 1985; Wong et al., 2002) and power calculation is not reported in any of the articles (Table 4). In only one article both genders were used (Stegenga & Kalb, 2001), in most studies (9/13) one single gender was used (Boyce et al., 1999; Luque et al., 1994; Nagy et al., 2004; Tolle et al., 1993, 1995; Verhovshek et al., 2005; Wee et al., 2008; Wong et al., 2002; Yung, 1998) and in three papers it was not reported (Table 4) (Brown et al., 2002; Kalb et al., 1992; Monaghan & Cotman, 1985). Blinding of the investigators was not reported in any of the articles (Table 4). In four papers, the quantification was performed in an objective manner by quantification with a computer or counting behind a microscope (Table 4) (Kalb et al., 1992; Monaghan & Cotman, 1985; Nagy et al., 2004; Verhovshek et al., 2005). The single western blotting study did not report whether correction for a housekeeping gene or protein concentration was performed (Table 4) (Brown et al., 2002). Eight articles were quantified in a subjective manner by observations of the investigators (Table 4) (Boyce et al., 1999; Stegenga & Kalb, 2001; Tolle et al., 1993, 1995; Wee et al., 2008; Wong et al., 2002; Yung, 1998). Furthermore, data reported on expression levels at different ages have not been duplicated with a similar study using the same technique. From this, we must be careful to draw firm conclusions based on the results of these papers.

**TABLE 4:** Quality assessment

Author	Year	1	2	3	4	5
		Number of animals	Power calculation	Use of both genders	Blinding	Objective quantification
Monaghan DT	1985	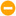	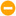	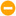	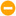	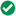
Kalb RG	1992	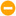	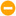	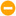	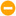	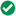
Tolle TR	1993	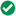	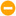	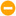	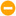	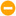
Luque JM	1994	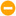	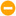	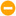	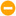	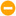
Tolle TR	1995	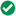	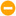	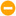	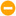	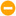
Yung KKL	1998	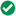	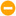	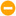	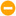	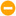
Boyce S	1999	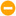	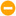	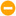	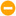	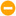
Stegenga SL	2001	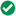	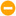	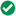	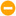	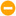
Brown KM	2002	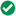	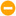	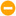	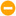	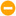
Wong HK	2002	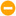	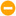	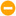	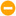	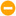
Nagy GG	2004	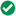	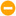	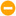	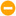	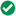
Verhovshek T	2005	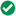	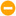	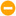	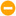	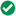
Wee KS‐L	2008	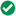	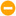	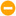	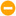	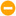

Notes

1: 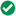 = number of animals used for expression data were reported 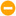 = Number of animals used was not reported 2. 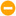 = it was not reported whether a power calculation was used to determine the number of animals needed. 3: 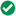 = both genders were used 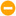 = only male or only female animals were used or it was not reported which gender was used. 4. 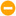 = Blinding of researchers or animal caregivers was not reported 5. 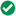 = expression levels were used in a standardized objective manner using predetermined tools 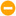 = quantification was performed in a subjective manner by researcher evaluation.

## 4 DISCUSSION

The results of this review on NMDAR expression in the spinal cord in rats indicates no change in total NMDAR from Birth (P0) to Adult in the dorsal horn (Brown et al., 2002; Kalb et al., 1992; Stegenga & Kalb, 2001). GluN1 splice variants are dynamically expressed in the dorsal horn during the development (Stegenga & Kalb, 2001). During the 1st week (P0–P7), the GluN1‐containing exon 22 is predominantly expressed (Stegenga & Kalb, 2001). During the second postnatal week (P8–P14) GluN1 splice variants containing exon 21 are described to be expressed more in this region (Stegenga & Kalb, 2001), while in the adult spinal dorsal horn GluN1 subunits containing exon 5 are expressed most (Luque et al., 1994; Stegenga & Kalb, 2001; Tolle et al., 1995).

Furthermore, based on our search we concluded that low to moderate levels of GluN2A, GluN2B, and GluN2C are present in the dorsal horn during the first postnatal week (P0–P7) (Stegenga & Kalb, 2001). These expression levels change to moderate levels of GluN2A and GluN2B in adulthood but then no GluN2C levels are noted (Boyce et al., 1999; Luque et al., 1994; Monaghan & Cotman, 1985; Nagy et al., 2004; Stegenga & Kalb, 2001; Tolle et al., 1993; Verhovshek et al., 2005; Yung, 1998). GluN3A and GluN3B are highly expressed in superficial layers of the dorsal horn in adult rats (Wee et al., 2008; Wong et al., 2002). There was no research data available on GluN4 subunit expression in the developing dorsal horn. Our risk of bias analysis shows that we must be careful to draw firm conclusions based on this data and that it is important to reproduce included research in a blinded objective manner. Unfortunately, our search yielded no information on NMDAR subunits on a cellular level. In the adult dorsal horn, NMDAR receptors are expressed both presynaptic on primary afferents and postsynaptic on second‐order neurons (Bardoni et al., 2004; Liu et al., 1994). This synaptic localization is important in the central sensitization process in which both presynaptic and postsynaptic NMDAR’s play an important role (Bardoni et al., 2004; Basbaum et al., 2009). The synaptic localization during the neonatal development is not known and, therefore, needs further research. Furthermore, NMDAR’s have been found in glial cells, however, it is unknown if these receptors contribute to nociceptive signaling (Žiak et al., 1998). Nevertheless, glial cells have an important role in nociception and chronification of pain (Beggs, 2011; Vallejo et al., 2010). Nociceptive input can activate astrocytes and microglia, which in turn release several cytokines, chemokines, and neuromodulators like ATP and BDNF (Beggs & Salter, 2013). These cytokines, chemokines,and neuromodulators can induce central sensitization within the spinal dorsal horn (Beggs et al., 2012; Trang et al., 2009). If NMDAR’s on microglia are present during the neonatal development and whether these contribute in nociceptive signaling or the development of the nociceptive system remains unknown. In order to unravel these gaps in knowledge, future research should include the use of advanced biochemical and/or imaging analysis techniques like PCR, Gene mapping, immune electron microscopy, or mass spectrometry.

The review of the data on the expression of the subunits and splice variants in the nociceptive system shows a dynamic expression pattern of GluN1 splice variants and NR2 subunits during the first two postnatal weeks of development in rats (Brown et al., 2002; Kalb et al., 1992; Stegenga & Kalb, 2001). As the NMDAR subunit and splice variant composition determines the functioning of the receptor, the question remains what effect this dynamic expression pattern may have on signal transduction and central sensitization processes (Ryan et al., 2008; Sugihara et al., 1992; Traynelis et al., 2010). GluN1 subunits expressing exon 5 have an increased current amplitude, while the lack of exon 5 increases NMDA affinity (Durand et al., 1993; Hollmann et al., 1993; Zhang et al., 1994). GluN1 subunits containing exon 21 have four serine phosphorylation sites, increasing the transport to the cell membrane and thereby increasing the expression, which may enhance central sensitization (Durand et al., 1993; Hollmann et al., 1993; Zhang et al., 1994). Splicing out of exon 22 increases protein kinase C (PKC)‐mediated potentiation (Tingley et al., 1993). PKC is a critical regulator of central sensitization, inclusion of exon 22 thus could reduce the central sensitization processes (Velázquez et al., 2007). This would imply that during the 1st week of development PKC‐mediated potentiation in the dorsal horn will be limited, while during the 2nd week this process and receptor phosphorylation would be enhanced, increasing susceptibility for central sensitization. During adulthood, NMDA affinity would then be reduced but current amplitude would be increased. Based on this information of GluN1 splice variants, the spinal dorsal horn is most susceptible to central sensitization during the 2nd week of postnatal development in rats.

The NMDAR is not only characterized by the GluN1 splice variants but NR2 subunits are crucial in determining the pharmacological and biophysical properties of the receptor (Cull‐Candy & Leszkiewicz, 2004). These NR2 subunits determine the affinity for glutamate, sensitivity to Mg^2+^ and channel kinetics (Cull‐Candy & Leszkiewicz, 2004). GluN2A‐containing receptors have a rapid deactivation kinetic, lower glutamate affinity with a strong Mg^2+^ block, and high conductance (Farrant et al., 1994; Momiyama et al., 1996; Vicini et al., 1998; Wyllie,et al., 1998). The GluN2B subunit‐containing receptors share this strong Mg^2+^ block and high conductance but have a higher glutamate affinity and slower deactivation kinetics, 250 ms versus 100 ms (Farrant et al., 1994; Momiyama et al., 1996; Vicini et al., 1998). The GluN2C‐containing receptors show a similar deactivation kinetic and glutamate affinity, but have a weak Mg^2+^ block and low‐conductance openings (35 and 18 vs. 50 picosiemens) (Farrant et al., 1994; Momiyama et al., 1996; Vicini et al., 1998). The GluN2D subunit‐containing receptors show a similar weak Mg^2+^ block, high glutamate affinity, and low‐conductance openings, but have an even slower deactivation kinetic of 4s (Farrant et al., 1994; Momiyama et al., 1996; Vicini et al., 1998; Wyllie et al., 1998). Furthermore, it was shown that the GluN2B is particularly involved in synaptic plasticity process as long‐term potentiation (LTP) and wind‐up, which are both processes similar to central sensitization (Crair, 1999; Dickenson & Sullivan, 1991). Based on the properties of the NMDA subunits, a higher expression of subunits GluN2C and or GluN2D would imply an increased neuronal excitability. For the postnatal development, this means that during the first 2 weeks there is a decrease in high excitable GluN2C subunits. Fast decaying and less sensitive GluN2A subunit‐containing NMDAR receptors and slower deactivating but more sensitive GluN2B receptors are dominant at mature synapses. Furthermore, GluN2A and GluN2B expression levels show a similar developmental expression pattern as both levels slightly increase during the first postnatal week (P0–P7). This is in contrast with findings in supraspinal brain areas but in line with recent findings on GluN2A and GluN2B contribution during development in the spinal cord (Liu et al., 2004; Mahmoud et al., 2020).

More recent work has been performed on the physiological aspects of NMDAR during the neonatal development in the spinal cord. These physiological and pharmacological studies imply that in the adult spinal dorsal horn NMDAR‐mediated nociceptive signaling is dominated by GluN2B and GluN2D (Hildebrand et al., 2014; Mahmoud et al., 2020). This finding is to some extend in contrast with the anatomical observations (see results section) which report a low expression of the GluN2D receptor. Obviously, a relative low expression of the GluN2D does not exclude a significant physiological role. In line these physiological findings an important role of GluN2B has been demonstrated in nociceptive signaling and central sensitization (Ji et al., 2003; Qiu et al., 2011). Electrophysiological experiments have shown that the presynaptic presence of this subunit at primary afferent neurons is important in the process of disinhibition, facilitating nociceptive input to the brain (Tong & MacDermott, 2014). Furthermore, during postnatal development (P1–P14), the expression of GluN2B was found to be regulated due to ubiquitination (Zhang et al., 2020). This regulation was effected by peripheral inflammation increasing the GluN2B expression (Zhang et al., 2020).

Overall, this may imply that rats will be more susceptible to central sensitization during the first two postnatal weeks (P0–P14). It should, however, be stressed that the number of papers investigating the NMDAR subunits and splice variant expression during postnatal development was low and no duplication of any study investigating the different time points in development was recorded (Brown et al., 2002; Kalb et al., 1992; Stegenga & Kalb, 2001). From a functional perspective both GluN2B and GluN2D seem the most important subunits in spinal nociceptive signaling throughout the neonatal development.

Study quality assessment showed that quantification of findings was often not performed in an objective manner and shows a high risk of bias in all studies included. In view of the average age of most studies included in this review, a high risk of bias is not unexpected as at that time standards for studies and statistics were not the same as today. This, however, does not imply that older articles are by definition more susceptible for bias. Furthermore, the effects of pain during the neonatal development on NMDAR subunit expression and electrophysiological response of the spinal dorsal horn have never been researched. In order to understand the role of NMDAR subunits and splice variants in nociception and central sensitization, further research is needed. This research could comprise; duplication of presented studies by objective quantitative analyses of NMDAR subunits and splice variants proteins. Furthermore, investigation on the effects of neonatal pain on the acute and adult NMDAR subunit expression would be a crucial next step; this could then be linked to central sensitization by electrophysiological recordings in the dorsal horn to painful and non‐painful stimuli.

### 4.1 Consequences for pain treatment in early life a premature and term newborn

Changes observed during the first postnatal week of the rat may have important consequences for translational research on nociception and pain, as the first postnatal week in rat is comparable to that of the third trimester of human neonates (Ian & Bryan, 2004; Ohmura & Kuniyoshi, 2017; Sikandar et al., 2013). Therefore, the first postnatal week in rat is used as a model for the situation in the neonatal Intensive care unit (NICU) (Knaepen, Rayen et al., 2013; Li et al., 2009; Schwaller & Fitzgerald, 2014; Van Den Hoogen et al., 2018; Walker, 2014). As premature newborns or ill infants admitted to a NICU are exposed to repetitive noxious and painful medical procedures, up to 14 times per day (Carbajal et al., 2008; Roofthooft et al., 2014) there is a major need for good mechanism‐based pain treatment. Hence, pain management in the NICU remains a major challenge (Allegaert & Van Den Anker, 2016; Fitzgerald & Walker, 2009). Neonatal exposure to pain and noxious stimuli results in acute hypersensitivity to pain but might also alter the neurodevelopment lasting in adulthood (Knaepen, Patijn et al., 2013; Van Den Hoogen et al., 2018; Walker, 2014). The fundamental process of central sensitization plays a crucial role in the acute and long‐lasting hypersensitivity in nociception and pain thus understanding and role of NMDA receptors is very important (LaPrairie & Murphy, 2009; Schwaller & Fitzgerald, 2014; Van Den Hoogen et al., 2018; Woolf, 2007). The results of this review indicate that important changes in NMDA subunit expression occur during the first two postnatal weeks of rats. Form a translational perspective this suggests that preterm and term born human neonates would be more susceptible to central sensitization processes. As the GluN2B and GluN2D receptor subunits are functionally most important in induction and maintenance of excitatory responses and thus for nociceptive signaling in neonatal spinal dorsal horn, specific antagonist directed to these subunits, GluN2B and GluN2D, are likely to be very useful and important targets to prevent both acute as well as long‐term consequences of neonatal pain.

In this context, the results of a recent study on neonatal procedural pain in rats with the use of methadone (subcutaneous injected 1 mg/kg) are very encouraging for clinical application of NMDA antagonists (Van den Hoogen et al., 2020). Methadone is not only a µ‐opioid antagonist but also a NMDA receptor antagonist (Ebert et al., 1995; Kristensen et al., 1994). Use of methadone during the first postnatal week (P0–P7) was able to not only prevent acute hypersensitivity but also reduced the long‐term effects on postoperative pain in the adult rat (Van den Hoogen et al., 2020). It must be noted that, early life treatment with methadone has shown to impact the cognitive development resulting in alterations of memory function (Chen et al., 2015; Schrott et al., 2008). In this review, we indicate that during the first 2 weeks expression of high excitable GluN2C decreases while expression of less excitable GluN2A and more sensitive GluN2B increases. Furthermore, we propose that excessive stimulation of the developing nociceptive system may alter this expression maturation. Therefore, specific NMDA subunit antagonist are promising candidates for mechanism‐based treatment of pain related to the developing nociceptive system as for instance in neonates.

In conclusion, this review shows that NMDAR subunits and splice variants are dynamically expressed during postnatal development from birth until adulthood in the spinal dorsal horn. The data suggest that, in rats, the nociceptive system is most susceptible to central sensitization processes during the first two postnatal weeks. From this, we conclude that targeted treatment directed toward specific NMDAR subunits is a promising candidate for mechanism‐based treatment of pain in neonates.

## CONFLICT OF INTEREST

All authors declare that they have no conflict of interest.

## Supporting information

Appendix A

## Data Availability

Data sharing is not applicable to this article as no new data were created or analyzed in this study, as this is a review of existing literature.
