# Supplementary material for: Qualitative review on N‐methyl‐D‐aspartate receptor expression in rat spinal cord during the postnatal development: Implications for central sensitization and pain
Source: Dev Neurobiol. 2020 Nov 20;80(11-12):443–55. doi: 10.1002/dneu.22789 (PMC7894158; doi:10.1002/dneu.22789)
Supplement: Supplementary file 1 — Appendix A [file DNEU-80-443-s001.docx]

***Appendix A***

Search terms and strategy per database:

Pubmed:

(((((((((((((expression[Title/Abstract]) OR protein expression[Title/Abstract]) OR protein level[Title/Abstract]) OR Immunohistochemistry[Title/Abstract]) OR electron microscopy[Title/Abstract]) OR Quantitative[Title/Abstract]) OR Western blot[Title/Abstract]) OR Localisation[Title/Abstract]) OR mRNA[Title/Abstract])) AND (((((Perinatal[Title/Abstract]) OR postnatal[Title/Abstract]) OR neonatal[Title/Abstract]) OR "Infant, newborn"[MeSH]) OR development[Title/Abstract])) AND ((("Receptors, N-Methyl-D-Aspartate"[Mesh]) OR "NR2B NMDA receptor"[Supplementary Concept]) OR "NR2A NMDA receptor"[Supplementary Concept]) OR "NR2D NMDA receptor" [Supplementary Concept]) OR "NR2C NMDA receptor" [Supplementary Concept]) OR NR3)[Title/Abstract] OR NR3A)[Title/Abstract] OR NR3B)[Title/Abstract] OR GluN3A)[Title/Abstract] OR GluN3B)[Title/Abstract] OR GRIN3A)[Title/Abstract] OR GRIN3B)[Title/Abstract] OR NR4)[Title/Abstract])) AND (((((Spinal cord[Title/Abstract]) OR Dorsal horn[Title/Abstract]) OR Spinal dorsal horn[Title/Abstract]) OR Substantia gelatinosa[Title/Abstract]) OR Laminae[Title/Abstract]))

Medline:

((n-methyl-d-aspartate receptor or nmda or nmdar or nr2a or nr2b or glutamate receptor epsilon 1 or GluRepsilon1 or NR2A N-Methyl-D-Asparate receptor or Glurepsilon2 or glutamate receptor epsilon 2 subunit or NR2B N-Methyl-D-Aspartate receptor or GRIN2B receptor or GLUN2B receptor or NMDA receptor 2B or NR2C or glutamate receptor epsilon 3 or GluN2C receptor or GluRepsilon3 or GluN2D receptor or NR2D or NR3 or NR3A or NR3B or GluN3A or GluN3B or GRIN3A or GRIN3B or NR4) and (Spinal cord or Dorsal horn or Spinal dorsal horn or Substantia gelatinosa or laminae) and (Perinatal or postnatal or neonatal or development or Newborn or Infants or Newborns or Newborn or Neonate or Neonates) and (expression or protein expression or protein level or Immunohistochemistry or electron microscopy or Quantitative or Western blot or Localisation or mRNA)).ab.

Embase:

((n-methyl-d-aspartate receptor or nmda or nmdar or nr2a or nr2b or glutamate receptor epsilon 1 or GluRepsilon1 or NR2A N-Methyl-D-Asparate receptor or Glurepsilon2 or glutamate receptor epsilon 2 subunit or NR2B N-Methyl-D-Aspartate receptor or GRIN2B receptor or GLUN2B receptor or NMDA receptor 2B or NR2C or glutamate receptor epsilon 3 or GluN2C receptor or GluRepsilon3 or GluN2D receptor or NR2D or NR3 or NR3A or NR3B or GluN3A or GluN3B or GRIN3A or GRIN3B or NR4) and (Spinal cord or Dorsal horn or Spinal dorsal horn or Substantia gelatinosa or laminae) and (Perinatal or postnatal or neonatal or development or Newborn or Infants or Newborns or Newborn or Neonate or Neonates) and (expression or protein expression or protein level or Immunohistochemistry or electron microscopy or Quantitative or Western blot or Localisation or mRNA)).ab.
